# Future Prospects of Metabolic and Bariatric Surgery: A Comprehensive Review

**DOI:** 10.3390/healthcare12171707

**Published:** 2024-08-26

**Authors:** Karl Hage, Gerardo Perrotta, Richard S. Betancourt, Jamil Danaf, Aryan Gajjar, Daniel Tomey, Katie Marrero, Omar M. Ghanem

**Affiliations:** 1 Department of Surgery, Mayo Clinic, Rochester, MN 55905, USA; betancourt.kylerichard@mayo.edu (R.S.B.); ghanem.omar@mayo.edu (O.M.G.); 2 Karsh Division of Gastroenterology and Hepatology, Cedars-Sinai Medical Center, Los Angeles, CA 90048, USA; 3 College of Medicine, Kansas City University, Kansas City, MO 64804, USA; jamil.danaf@kansascity.edu; 4 Department of Surgery, David Geffen School of Medicine, University of California, Los Angeles, CA 90095, USA; agajjar1@g.ucla.edu; 5 Department of Surgery, Houston Methodist Hospital, Houston, TX 77030, USA; datomey@houstonmethodist.org; 6 Carle Foundation Hospital General Surgery Residency, Champaign, IL 61801, USA; kam13@illinois.edu

**Keywords:** metabolic surgery, bariatric surgery, obesity, gastric bypass, sleeve gastrectomy

## Abstract

Background: The field of metabolic and bariatric surgery (MBS) is currently an expanding surgical field with constant refinements in techniques, outcomes, indications, and objectives. MBS has been effectively applied across diverse patient demographics, including varying ages, genders, body mass indexes, and comorbidity statuses. Methods: We performed a comprehensive literature review of published retrospective cohort studies, meta-analyses, systematic reviews, and literature reviews from inception to 2024, reporting outcomes of MBS using databases such as PubMed, ScienceDirect, and Springer Link. Results: MBS is a safe and efficient therapeutic option for patients with obesity and associated medical conditions (mortality rate 0.03–0.2%; complication rates 0.4–1%). The favorable safety profile of MBS in the short-, mid-, and long-term offers the potential to treat patients with obesity and type 2 diabetes mellitus, immunosuppression, chronic anticoagulation, neoplastic disease, and end-organ failure without increased morbidity and mortality. Conclusions: In conclusion, the future of MBS lies in the ongoing innovation and adapted therapeutic strategies along with the integration of a variety of other techniques for managing obesity. Careful preoperative assessments, coupled with a multidisciplinary approach, remain essential to ensure optimal surgical outcomes and patient satisfaction after MBS.

## 1. Introduction: A Brief History of Metabolic and Bariatric Surgery

The chronic disease of obesity has emerged as a significant global health concern, impacting individuals across diverse age groups, genders, and socioeconomic statuses. Its increasing prevalence can be attributed to various factors, including sedentary lifestyles, poor dietary choices, genetic predisposition, and environmental impact. Recent epidemiological studies indicate that approximately one in eight individuals, or roughly 1 billion people worldwide, are affected by obesity [1]. This condition is not only a standalone health issue but also a pivotal factor in the development and progression of multiple closely linked medical conditions, such as type 2 diabetes mellitus (T2DM), cardiovascular diseases, and certain cancers [2]. Additionally, the consequences of obesity extend beyond physical health, also impacting mental well-being and quality of life [3].

The field of obesity treatment has witnessed a significant evolution, providing patients with modified and improved options since the mid-20th century. Therapeutic options include behavioral and lifestyle modifications, medical management, and surgical management via metabolic and bariatric surgery (MBS). Initially focused on restricting food intake or bypassing specific sections of the small bowel, early MBS procedures, such as the jejunoileal bypass, faced important challenges, including high rates of liver failure and other complications [4]. In 1960, Dr. Edward Mason introduced the gastric bypass, marking a significant advancement in MBS, with reduced complication rates and improved patient outcomes. The gastric bypass was a pioneering effort that led the way for subsequent modern MBS procedures, including laparoscopic approaches introduced in the early 1990s [5]. The late 1990s and early 2000s witnessed the rise of MBS procedures, including the sleeve gastrectomy (SG) and duodenal switch, gaining popularity due to their efficacy in weight loss outcomes and enhanced quality of life reported by patients. The growth of the MBS field is reflected by the increase in numbers of performed procedures annually with an overall total of 280,000 in 2022 in the United States [6]. This substantial growth alludes to the future prospects of MBS and its application in different medical fields.

In this study, we aim to elaborate on some of the future prospects of MBS by debunking the myth regarding increased morbidity and demonstrating that MBS is an efficient short-, mid-, and long-term therapeutic option for patients with obesity, irrespective of age, gender, immunosuppression status, and other co-morbid medical conditions [7,8]. Additionally, it is essential to discuss the role of MBS in patients with end-stage organ failure as a bridge to organ transplant [9]. Overall, the future of MBS lies in the ongoing innovation and adapted therapeutic strategies along with the integration of a variety of other techniques for managing obesity.

## 2. Materials and Methods

**Figure 1:**
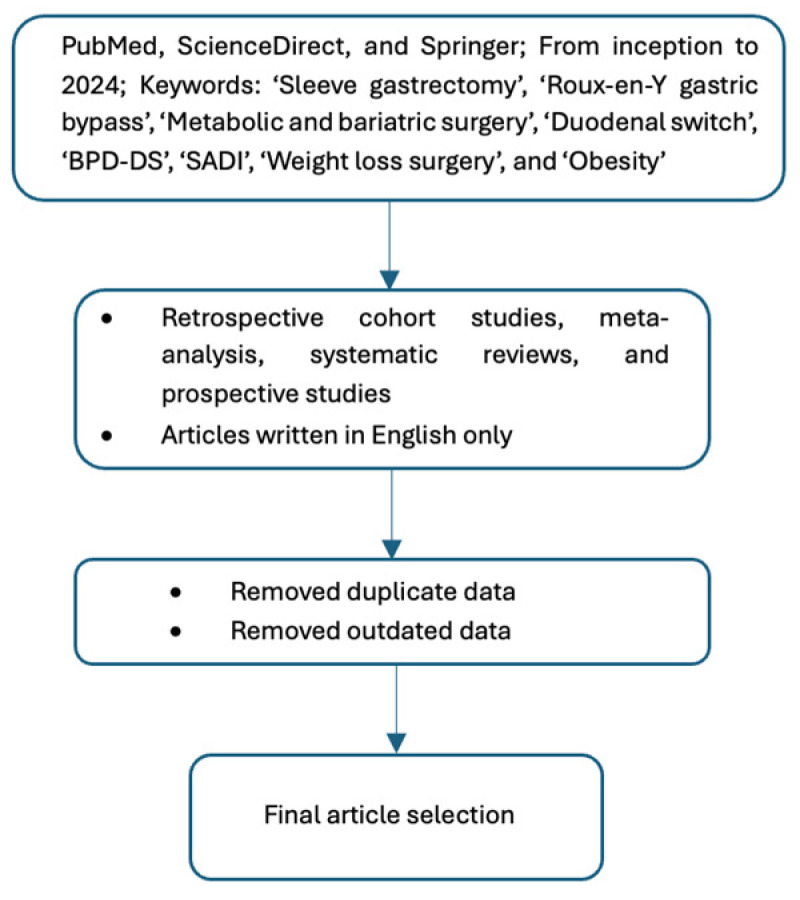
Article selection flowchart.

The authors of this manuscript conducted a comprehensive narrative literature review (LR) through a structured process. This involved defining relevant research questions and keywords, conducting the LR, and summarizing research findings. All authors collaborated on various tasks, including drafting an LR protocol, identifying inclusion and exclusion criteria, establishing a suitable search strategy, selecting appropriate search engines, ensuring the quality of selected articles, and extracting relevant data for the final manuscript. Retrospective cohort studies, meta-analyses, systematic reviews, and prospective studies reporting outcomes of MBS in English were included in our study selection. Exclusion criteria included abstracts, non-English texts, and papers that do not include our relevant search words such as ‘Sleeve gastrectomy’, ‘Roux-en-Y gastric bypass’, ‘Metabolic and bariatric surgery’, ‘Duodenal switch’, ‘BPD-DS’, ‘SADI’, ‘Weight loss surgery’, and ‘Obesity’. Relevant articles from inception to 2024 were identified using databases such as PubMed, ScienceDirect, and Springer Link, and filtered based on specific keywords. Manuscripts were screened and duplicates, articles that did not meet our inclusion criteria, as well as articles that presented outdated data, were removed. The corresponding author approved the final selection of articles and expert opinion provided by the senior author was used to synthesize the gathered information and edit the manuscript draft (Figure 1 and Supplementary Table S1).

## 3. Results

### 3.1. The Safety of MBS: Debunking the Myth of Increased Morbidity

MBS has evolved over the past two decades, leading to significant improvements in safety and long-term outcomes [10]. Although MBS is the most effective treatment for obesity and obesity-related medical conditions [11,12], many physicians are still reluctant to widely offer surgery to their patients due to concerns about associated risks and morbidity [13,14]. This applies particularly to more novel bariatric procedures such as the duodenal switch. Despite the significant increase in MBS procedures in the United States from 8631 in 1993 to 162,969 in 2017 [1], it remains underutilized, being offered to only 1% of the eligible population [15]. Overall, MBS complication and mortality rates peaked in 1998 (11.7% and 1%, respectively), and then steadily decreased throughout 2016 (1.4% and 0.4%) [10]. In 2020, long-term mortality rates were reported as low as 0.03–0.2% [11,16].

The two most common MBS procedures [sleeve gastrectomy (SG) and Roux-en-Y gastric bypass (RYGB)] have been extensively studied recently, showing a favorable trend towards decreased rates of short-term morbidity and mortality, including a decreased risk of deep vein thrombosis/pulmonary embolism (DVT/PE), staple line or anastomotic leak, surgical site infection, and the need for reoperation [17,18], all of which were more significant in the past. Some short-term minor complications still occur in 1.16–4.94% of all cases [19]. Regarding long-term complications, RYGB is generally associated with higher rates of postoperative bleeding, marginal ulcers, stenosis, and reoperations compared to SG [20]. Other long-term complications include protein–calorie malnutrition and vitamin deficiencies [21]. The development or worsening of pre-existing GERD remains the most frequent long-term complication after SG [22].

Factors that have contributed to the decreased incidence of severe complications following MBS include improved patient selection, refined surgical techniques, and better training in perioperative care [23]. Not only has MBS become much safer over the past 20 years, but its short-term safety profile has now become comparable to that of other common abdominal/pelvic procedures that are considered low risk. One study analyzed data from 1.6 million patients undergoing MBS or common abdominal/pelvic procedures [24] and showed that serious complications following MBS procedures were exceedingly rare, as rates of DVT/PE (0.5% of all cases), pneumonia (0.3%), urinary tract infections (0.6%), and sepsis (0.4%) were among the lowest of any procedure [24]. Additionally, the most common causes of readmission in MBS patients were nausea, abdominal pain, and dehydration, all of which are relatively minor and easily manageable [25]. Lastly, mortality rates (0.1%) were comparable to that of laparoscopic ventral hernia repair and Nissen fundoplication, and lower than laparoscopic colectomy and laparoscopic total abdominal hysterectomy and bilateral salpingo-oophorectomy (TAHBSO) [24].

Two key developments have also influenced the positive trend of improved safety after MBS: the adoption of the Enhanced Recovery After Bariatric Surgery (ERABS) protocols and the Metabolic and Bariatric Surgery Accreditation and Quality Improvement Program (MBSAQIP). These protocols aim to define and implement a standard of care to improve surgical outcomes. Since 2015, multiple studies demonstrated improved outcomes, reduced mortality, and readmission rates in centers adopting ERABS protocols [26,27]. Similarly, after its institution in 2012, the MBSAQIP accreditation provided institutions with standards of practice and human resources to optimize surgical outcomes [28], leading to a decrease in complication rates from 6% to 3% in accredited centers [29,30]. This provides ample data to better understand the current state of MBS, but also gives a path forward to continually improve the efficacy and safety profiles of MBS procedures, thus debunking the myth regarding increased morbidity for patients with obesity.

### 3.2. Is Age a Limitation for MBS?

As the understanding of obesity and the treatment therein advances, the indications for MBS have adapted as well. The guidelines established in 1991 by the National Institutes of Health (NIH) did not recommend MBS for children and adolescents under the age of 18 due to a lack of sufficient information concerning its efficacy and health outcomes [31]. Additionally, a 1998 evidence report from the NIH stated that they were unable to conclude whether the surgical treatment of obesity was effective in patients after the age of 60 or 65 years [32]. However, due to the recent substantial increase in the prevalence of obesity in pediatric and older adult populations, as well as the expanding number of older adults in the general population, these recommendations have been amended to offer MBS more broadly to patients at the extremes of age, including patients younger than 18 years old or adult patients aged 70 years and older. A report by the American Medical Association (AMA) in 2013, recognizing obesity as a global epidemic, also drove the investigation of MBS’s efficacy and safety in pediatric and elderly populations [33].

Following recent advancements in MBS procedures which were associated with improved outcomes, higher weight loss, and a more favorable safety profile, indications from the American Society for Metabolic and Bariatric Surgery (ASMBS) and the International Federation for the Surgery of Obesity and Metabolic Disorders (IFSO) have emerged and changed the patient selection parameters for MBS to include pediatric and elderly populations [31].

#### 3.2.1. Pediatric Population

Regarding the pediatric population, the current guidelines from the ASMBS and American Academy of Pediatrics (AAP), reported in 2018 and 2019, respectively, demonstrate MBS to be safe in patients under 18 years of age [34,35]. Similar to the adult population, a multidisciplinary team approach is recommended for preoperative decision making and postoperative care. The contraindications presented for adolescents, those aged between 10 and 18 [36], include having a medically correctable cause of obesity, the presence of ongoing substance abuse, conditions that would restrict the adherence to postoperative care, as well as current or planned pregnancy within 12 to 18 months of the procedure. In relation to the type of procedure, SG was specified as the currently preferred procedure in adolescents. A 2023 multicenter randomized control trial in Sweden comparing surgical and non-surgical treatment outcomes for obesity in adolescent patients aged 13 to 16 years old demonstrated a significantly higher weight loss and comorbidity resolution in the surgical group while there was no difference in safety, hospitalization, and mortality rates between the two groups [37]. Multiple other retrospective studies validate consistent findings that MBS is a safe and efficient therapeutic option for pediatric patients with obesity after a thorough assessment and using a multidisciplinary approach to optimize surgical outcomes [38]. Moreover, a recent study using the national Metabolic and Bariatric Surgery Accreditation and Quality improvement Program (MBSAQIP) database stratified the cohorts into three categories: adolescents (13–17 years of age), college-aged (18–21), and young adults (22–25). Results showed that postoperative complications rates were similar between age groups at 1% [39]. Lastly, in addition to recognizing its impact on physical health, the postoperative outcomes as a result of MBS have been found to have a positive effect on mental health in adolescents. A systematic review of 20 studies reported significant and sustained improvement in psychosocial outcomes after MBS in patients aged 18 years and younger [40].

#### 3.2.2. Elderly Population

Regarding MBS in older adults, the 2022 ASMBS report indicates that MBS provides appreciable weight loss outcomes and comorbidity resolution, despite showing a slightly higher complication rate compared to younger patients. Currently, there is no upper age limit indicated for surgery, given the variance in the aging process between individuals. A preoperative evaluation of patient frailty during the patient selection process is recommended [41], and age alone should not be a barrier to MBS. Unlike the pediatric population, the elderly population was not given clear recommendations for procedure preference between RYGB and SG.

One retrospective study compared the short-term safety and efficacy of MBS between RYGB and SG in patients ≥ 65 years old and a < 65 years old control group. There was no statistically significant difference between procedure type or short-term health outcomes of hospital length of stay (LOS), 1-month BMI change, and complication rate [42]. Concerning the resolution of comorbidities, RYGB demonstrated a greater degree of resolution in sleep apnea and T2DM compared to SG [43]. From these studies, the safety of MBS in the elderly population was shown to be similar to the general population. While SG and RYGB had similar long-term weight loss outcomes, the superior resolution in comorbidities in patients undergoing RYGB may be a relevant factor to consider during procedure selection.

Lastly, while MBS alone has been shown to be safe in elderly patients, more investigations into the safety of MBS with concomitant procedures is needed for this specific population. One study from 2023 investigated the early and long-term outcomes of older adults undergoing paraesophageal hernia repair and RYGB simultaneously to evaluate this as a surgical option. Careful preoperative assessments and functional status were shown to be important parameters to consider during patient selection to ensure optimal outcomes and reduce postoperative morbidity and mortality [44].

### 3.3. Effect of Preoperative BMI on MBS Outcomes

Despite recent advancements in the field of MBS, operating on patients with very high BMIs (>50–60 kg/m^2^) remains challenging due to the higher risks of perceived complications in this patient population. A high preoperative BMI is correlated with increased technical difficulties as well as a higher burden of disease, which is a known predictive factor of higher complication rates. However, recent reports have consistently demonstrated that MBS appears to be safe and efficient in patients with a preoperative BMI ≥ 50 kg/m^2^ [45,46,47]. To further advance the investigation on the impact of preoperative BMI, multiple efforts were made to report outcomes of MBS in patients with a BMI in the 60 and 70 kg/m^2^ range. Badaoui et al. compared surgical outcomes between patients with a BMI ranging from 50 to 60 kg/m^2^ and patients with a BMI > 60 kg/m^2^ and demonstrated that weight loss outcomes were comparable in the long-term (5 years) and that there were no differences in complication rates after controlling for certain confounders [45]. This study provided evidence supporting the similar management of patients despite specific subgroups of BMI to obtain favorable outcomes after MBS.

Regarding MBS outcomes in patients with a preoperative BMI ≥ 70 kg/m^2^, there remains a scarcity of published literature. Nevertheless, Romero-Velez et al. noted longer operative times and hospital stays in patients with a BMI ≥ 70 kg/m^2^ compared to those with lower preoperative BMI, and a 30-day mortality that was slightly higher in the higher BMI group, although it remained relatively low (0.4% vs. 0.1%) [48].

Despite the technical challenges associated with a very high BMI, the efficacy of MBS in this patient population remains high. In a comparative study between biliopancreatic diversion with duodenal switch (BPD-DS), RYGB, and SG in patients with BMI ≥ 70 kg/m^2^, BPD-DS was found to achieve the highest total weight loss (%TWL) at 24 months (40.6% vs. 33.8% in the RYGB group, and 28.5% in the SG group), with similar rates of comorbidity resolution and complications across the three cohorts [49]. Similar results were obtained in patients with a BMI ≥ 50 kg/m^2^ with a longer follow-up of up to 60 months [50]. SADI-S [51] and OAGB [52] were also found to be particularly effective in patients with a very high BMI, and were therefore recommended, together with RYGB, by a group of experts in a recent Delphi consensus [53].

### 3.4. MBS and Type 2 Diabetes Mellitus Management

With the constant refinement of MBS procedures, the role of weight loss surgery in the management of multiple obesity-related medical conditions such as hypertension, obstructive sleep apnea, dyslipidemia, and others, has been extensively studied. A significant area of interest in bariatric surgery is the sustained effect of MBS on the remission of type 2 diabetes mellitus (T2DM) in patients with obesity. The controversy on this matter stems from the fact that MBS was generally avoided in the past for the treatment of T2DM due to the associated postoperative complications [54]. In fact, it was not before 2011 that the International Diabetes Federation Taskforce on Epidemiology and Prevention of Diabetes approved a consensus decision between diabetologists, endocrinologists, and surgeons regarding the appropriate role of MBS in the treatment and prevention of T2DM [54]. Other therapeutic options for patients with T2DM and obesity include behavioral and dietary modifications as well as anti-obesity medications. Multiple recent meta-analyses have demonstrated the significantly superior results of MBS in achieving complete and prolonged T2DM remission compared to other therapeutic options [55,56]. Indeed, less than 10% of patients achieve T2DM remission with a strict behavioral, dietary, and exercise program alone [55]. Similarly, these rates only reach up to 30% using anti-obesity medications such as GLP-1 agonists and others [57]. On the other hand, T2DM remission can be achieved with rates up to 40–50% after SG, 50–60% after RYGB, and even 80–90% after duodenal switch procedures (BPD-DS and SADI-S) [2,58]. Recent results also demonstrated that the longer preoperative duration of T2DM was directly correlated with lower T2DM remission rates after MBS, with an annual decrease of 7% in T2DM remission rates for each year of delay to undergo surgery [59]. Additionally, emerging studies showed that patients who undergo MBS can experience a continued diabetes remission despite weight recurrence, indicating that the mechanism of action for T2DM remission extends beyond pure weight loss and is affected by hormonal changes following MBS [60]. Nevertheless, it is important to acknowledge that multiple patient-specific variables such as preoperative T2DM severity, reflected by insulin use, duration of T2DM, and HbA1c level, as well as postoperative weight loss can affect the results of MBS [2]. For this reason, specific remission scores have been established and are currently being used by physicians to facilitate the procedure selection choice but also incentivize patients to adhere to a close postoperative follow-up and optimize their weight loss after MBS [61,62,63].

### 3.5. MBS for Patients on Long-Term Anticoagulation

With the steady increase in age and obesity-related medical conditions in patients with obesity, one of the challenges of MBS is its safety in high-risk populations, such as those taking anticoagulant medications. Approximately 3% of individuals undergoing MBS are on chronic anticoagulation therapy (CAT) [64], defined as treatment with either Warfarin or a Direct Oral Anticoagulant (DOAC) for at least 90 days before surgery. Co-existing medical conditions such as atrial fibrillation, previous venous thromboembolism, the presence of a mechanical cardiac valve, or thrombophilia justify the need for CAT in this patient population.

Multiple studies have investigated the incidence of postoperative complications in patients on CAT following MBS. The 30-day bleeding rate in the immediate postoperative period has been reported at 3.78%, compared to 0.88% in patients undergoing MBS and who are not anticoagulated (*p* < 0.001). The overall 30-day major complication rate is higher in patients on CAT (8.73% vs. 3.36%, *p* < 0.001) [65], with RYGB patients experiencing more complications compared to those undergoing SG. In a different cohort with a much longer follow-up of 15 years, the incidence of long-term bleeding (>30 days postoperatively) was found to be significantly higher in patients undergoing RYGB (18.3%), compared to 4% of patients undergoing SG (*p* = 0.017) [66]. Bleeding was also more likely in patients treated with Warfarin compared to DOAC, and in those with concurrent antiplatelet therapy (high-dose Aspirin or Clopidogrel). The majority of bleeding episodes in the RYGB group were due to marginal ulcers, a well-known complication of this procedure with an incidence of up to 25% [67].

These findings highlight the importance of close patient monitoring not only in the immediate postoperative period, but also on the long-term as well as the need for appropriate patient counselling regarding the individual bleeding risk after MBS. Nevertheless, these results do not imply that MBS should be denied to patients on CAT but serve as a forewarning to ensure surgical safety.

### 3.6. MBS and Neoplastic Disease

The exact mechanisms of cancer development in patients with obesity have not been clearly identified yet. For hormonal cancers, the excessive adiposity may contribute to accelerating the neoplastic process due to the chronic state of inflammation and release of adipokines and sex steroid hormones. Insulin resistance is also another potential cause of cell mutation for this specific cohort of patients [68].

Endometrial and breast cancer have the strongest association with obesity, as multiple retrospectives studies showed that MBS can efficiently reduce the obesity-associated risks of developing cancer. However, establishing a strong correlation between weight loss surgery and hormonal cancer risk reduction remains difficult due to the relatively low incidence of disease and the long follow-up period required to establish these conclusions. In a recent large cohort study, Aminian et al. matched adult patients with obesity undergoing MBS (either RYGB or SG) to a non-surgical cohort and reported the incidence of obesity-associated cancer and cancer-related mortality after a mean follow-up period of 6.1 years. The results demonstrated that the cohort of patients who underwent MBS had a significantly lower incidence of endometrial and breast cancer compared to the non-surgical group [2.9% vs. 4.9% (95% CI, 2.2–3.6%)] as well as a lower overall cancer-related mortality [0.8% vs. 1.4% (95% CI, 0.4–1.2%)] [69].

Additionally, the effect of MBS on cancer risk reduction applies to non-hormonal cancers such as esophageal, gastric, liver, gallbladder, colorectal, pancreatic, and kidney cancer [70]. The published literature on this topic remains scarce; however, the positive effect of weight loss surgery, coupled with a close postoperative multidisciplinary approach, has demonstrated favorable results for patients with obesity and cancer. An interesting result to mention from Jawhar et al.’s study is that AGB was not associated with a reduction in cancer risk, unlike the other two commonly performed operations, RYGB and SG [70,71]. This implies that the impact of MBS on cancer risk reduction extends beyond pure restrictive weight loss, as seen with AGB, and could potentially be due to the anatomical and hormonal changes seen with other MBS procedures. Lastly, MBS also appears to play a significant role as a bridge to neoplastic therapy in patients with an established diagnosis of cancer. A multi-centered study on thirty-seven patients with nine distinct organs of origin of primary low-grade neoplasia showed a significant BMI reduction after MBS, allowing 84% of patients to undergo successful neoplastic surgery without any MBS-associated mortality [9].

### 3.7. MBS in Patients with Immunosuppression

The success of MBS relies significantly on the effective postoperative healing process of gastrointestinal structures, which has been shown to impact patient quality of life. However, this healing process can be negatively affected by chronic steroid use, immunosuppressants, and inflammatory conditions. The effect of chronic immunosuppression on surgical outcomes have been well documented in other surgical fields. This translates into an inability to properly heal the anastomoses created during the MBS procedures. Unfortunately, the published literature still lacks sufficient data to validate the association between chronic immunosuppression and negative MBS outcomes [72].

In an effort to elucidate this association, Maroun et al. reported the short-, mid-, and long-term outcomes of chronically immunosuppressed patients undergoing RYGB, SG, and BPD-DS. The rate of intra-operative complications was relatively low at 2%, while the early (<30 days) complication rate was higher at around 17%. Nevertheless, these complications were classified as minor in nature (UTI, pneumonia, and superficial surgical site infections) and were managed successfully without any impact on long-term morbidity and mortality. Regarding long-term complications, the most common occurrences were marginal ulcers following RYGB and SBO (both 3.3%) [72]. Another larger scale study, using the national MBSAQIP database, aimed to compare outcomes between immunocompetent and immunosuppressed patients undergoing MBS. They demonstrated a significantly higher major complication rate at the 30-day follow-up period, especially following RYGB (OR 1.39, 95% CI 1.25–1.55; *p* < 0.001). These rates were primarily driven by a higher incidence of postoperative bleeding (OR 1.49, 95% CI 1.24–1.80; *p* < 0.001) and anastomotic leak (OR 1.38, 95% CI 1.02–1.87; *p* = 0.037) in the immunosuppressed patient cohort. The 30-day mortality, however, was comparable between the two groups [73].

These results shed light on the outcomes of MBS in a very specific patient population with significant risk factors. The lack of further data should be driving additional research efforts to elucidate the exact association between immunosuppression and MBS outcomes. However, at this time, it appears that MBS is a relatively safe and efficient long-term option for immunosuppressed patients with relatively low and minor complication rates.

### 3.8. MBS and End-Organ Disease: A Bridge to Solid Organ Transplantation

In the United States alone, there is currently over 100,000 patients with end-organ failure requiring kidney, liver, heart, and pulmonary transplantation, with only a minority that are actively being taken off the waiting list [74]. With obesity being a main limiting factor for patients seeking transplantation, it becomes crucial to offer this specific patient population a sustained and efficient therapeutic option to achieve the ideal preoperative BMI of <35 kg/m^2^ and become eligible for transplantation [75,76,77].

Patients with obesity and end-stage organ failure constitute a particular subset of patients who will eventually be started on steroid medication and immunosuppressants following transplantation. For this reason, physicians are reluctant to offer MBS to avoid potential complications such as staple line leaks, marginal ulceration, malnutrition, and malabsorption [78]. Nevertheless, emerging studies have repeatedly validated the favorable outcomes associated with MBS as a bridge to solid organ transplantation [75,79,80]. MBS outcomes are most commonly studied in the setting of kidney transplantation with multiple reports describing a substantial increase in successful weight loss, transplant eligibility, access to kidney transplantation, and long-term graft survival for patients with end-stage renal disease undergoing weight loss surgery. A recent meta-analysis of 21 studies demonstrated that the pooled rate of patients who were successfully listed for kidney transplant reached 83% (95% [CI] 57–99) after MBS, with 83% (95% CI 65–97) of patients subsequently receiving successful transplantation. The 30-day complication rate in this patient cohort was 0.5%, which is comparable to the rates seen in the general bariatric population [81]. Similarly, patients awaiting liver transplantation can vastly benefit from the weight loss effects of MBS to increase eligibility and transplant success rates. End-stage liver failure and obesity are two very closely linked diseases, with 23% of liver transplant candidates having a BMI > 30 kg/m^2^, 10% having a BMI > 35 kg/m^2^, and 4% having a BMI of 40 kg/m^2^ or higher [82]. Once a target BMI of <35 kg/m^2^ is achieved with MBS, liver transplant rates have been reported to reach up to 70–80%, with a complication rate of less than 5% [83].

MBS, in synergy with left ventricular assist device (LVAD) implantation, in the scope of end-stage heart failure management, has shown that 67.4% of patients are listed on the transplant waitlist, with 32.5% undergoing successful heart transplant after MBS [84]. Despite the one-year mortality reaching up to 10% in this study, it is important to remember that this specific patient population is at a particularly high risk, and these rates are still lower than the mortality rates of LVAD implantation as a destination therapy alone, which can reach up to 48% at the one-year mark [85]. Therefore, MBS is justified as a bridge to heart transplantation to increase transplant eligibility and ensure optimal surgical outcomes.

In terms of preferred procedure, SG seems to be the superior option due to its lower technical complexity in an already complex patient population. The only exception appears to be patients undergoing lung transplantation, where RYGB is preferred over the SG to reduce the risks of postoperative reflux and associated micro aspirations that could potentially negatively impact the graft [86]. MBS for patients awaiting organ transplants requires careful physiologic, pharmacologic, and psychological considerations and should be offered in the setting of a multidisciplinary approach with a personalized care plan to each patient with obesity not meeting the requirements for transplantation.

### 3.9. MBS as a Bridge for Other Destination Procedures

Increased body mass is a known risk factor for multiple diseases. In this setting, MBS offers the potential to reduce patients’ weights and bridge them for other destination procedures.

Abdominal wall hernia (AWH) repair in patients with obesity, in association with MBS, requires a careful consideration of physiologic and technical components for optimal outcomes. The treatment of an AWH during MBS can either be performed in the immediate/concurrent setting or in a delayed two-step approach. Either way, the weight loss benefit of MBS is associated with significantly higher AWH repair success rates and a lower technical complexity, which translates into a reduction of postoperative complications [87]. The short-term hernia recurrence rate is reported to be 2–5% after a combined approach of AWH repair and MBS, compared to 10–14% in patients who did not undergo MBS [88].

Obesity is also associated with the worsening and progression of multiple common gynecologic pathologies such as endometriosis, dysmenorrhea, uterine fibroids, abnormal uterine bleeding, endometrial hyperplasia, and gynecologic cancers. Given that 15% of patients who undergo therapeutic hysterectomy experience postoperative complications, prior MBS and weight loss could be beneficial to mitigate these complications and improve patient outcomes [89]. A recent study using the National Inpatient Sample (NIS) database matched patients who underwent a hysterectomy with a prior history of MBS with another group of patients undergoing a hysterectomy without prior MBS to evaluate for postoperative outcomes. Interestingly, after controlling for specific preoperative parameters such as age, BMI, and comorbidity scores, patients with a previous history of MBS experienced fewer complications [OR 1.048 (1.06–1.09); *p* < 0.001] and shorter lengths of stay (0.841 vs. 0.906; *p* = 0.02) after hysterectomy compared to their counterparts without a history of MBS [90].

The effect of BMI reduction after MBS also applies to different medical fields including rheumatology, dermatology, orthopedic surgery, and more. Further research into these specific patient populations is required to support the important role of MBS as a bridge to other destination procedures.

## 4. Conclusions

The field of MBS is currently experiencing the ongoing development and refinement of procedures, techniques, indications, patient considerations, and objectives. A once previously underperformed and underutilized approach, MBS has gained significant popularity and now offers efficient therapeutic options to patients with obesity and additional related conditions such as T2DM, immunosuppression, CAT, neoplastic disease, end-organ failure, and others. Physicians should be cognizant that a careful patient assessment coupled with a multidisciplinary approach remains essential to ensure optimal surgical outcomes and patient satisfaction after MBS.

## Data Availability

Data can be provided upon reasonable request from the corresponding author.

## Supplementary Materials

The following supporting information can be downloaded at: https://www.mdpi.com/article/10.3390/healthcare12171707/s1, Table S1: Summary of the included studies.
