# Supplementary material for: Future Prospects of Metabolic and Bariatric Surgery: A Comprehensive Review
Source: Healthcare (Basel). 2024 Aug 26;12(17):1707. doi: 10.3390/healthcare12171707 (PMC11395476; doi:10.3390/healthcare12171707)
Supplement: Supplementary file 1 [file healthcare-12-01707-s001.zip › healthcare-3131109-supplementary.pdf]

First Author	Year of Publication	Country of Publication	Type of Study
Hage K (2)	2023	USA	Retrospective Cohort
Segal Y (3)	2024	USA	Review Article
Faria GR (4)	2017	Portugal	Review Article
O'Brien P (5)	2000	Australia	Review Article
Clapp B (6)	2024	USA	Retrospective Cohort
Fischer L (7)	2015	Germany	Randomized Controlled Trial
Talerico R (8)	2024	Italy	Review Article
Parmar C (9)	2024	USA	Retrospective Cohort
Campos GM (10)	2020	USA	Retrospective Cohort
Arteburn DE (11)	2020	USA	Review Article
Billeter AT (12)	2018	Germany	Meta-analysis
Lopez EKH (13)	2020	USA	Survey
Clapp B (14)	2021	USA	Survey
Campos GM (15)	202	USA	Retrospective Cohort
English WJ (16)	2018	USA	Retrospective Cohort
Clapp B (17)	2022	USA	Retrospective Cohort
Khan S (18)	2016	USA	Retrospective Cohort
Hage K (19)	2024	USA	Retrospective Cohort
Woźniewska P (20)	2021	Poland	Review Article
Lim R (21)	2018	USA	Retrospective Cohort
Auge M (22)	2022	France	Retrospective Cohort
Bevilacqua LA (23)	2020	USA	Retrospective Cohort
Kearns EC (24)	2021	USA	Retrospective Cohort
Clapp B (25)	2024	USA	Retrospective Cohort
Aman MW (26)	2016	USA	Retrospective Cohort
Agnoletti V (27)	2020	Italy	Retrospective Cohort
Małczak P (28)	2017	USA	Meta-analysis
Carter J (29)	2024	USA	Retrospective Cohort
Al-Mazrou AM (30)	2023	USA	Retrospective Cohort
Clapp B (31)	2022	USA	Retrospective Cohort
Eisenberg D (32)	2022	USA	Guideline Paper
Pratt JSA (35)	2018	USA	Guideline Paper
Armstrong SC (36)	2019	USA	Retrospective Cohort
Järvholm K (38)	2023	Sweden	Randomized Controlled Trial
Ahmed AE (39)	2019	Qatar	Retrospective Cohort
Grant HM (40)	2021	USA	Retrospective Cohort
Trooboff SW (41)	2019	USA	Meta-analysis
Mrdutt MM (42)	2019	USA	Retrospective Cohort
Vinan-Vega M (43)	2019	USA	Retrospective Cohort
Belluzzi A (44)	2023	USA	Retrospective Cohort
Hage K (45)	2023	USA	Retrospective Cohort
Badaoui JN (46)	2022	USA	Retrospective Cohort
Prachand VN (47)	2006	USA	Retrospective Cohort

Parikh MS (48)	2005	USA	Retrospective Cohort
Romero-Velez G (49)	2020	USA	Retrospective Cohort
Abi Mosleh K (50)	2024	USA	Retrospective Cohort
Maroun J (51)	2022	USA	Retrospective Cohort
Lind RP (52)	2022	USA	Retrospective Cohort
Parmar CD (53)	2019	USA	Retrospective Cohort
Ponce de Leon-Ballesteros G (54)	2024	USA	Retrospective Cohort
Mirghani H (56)	2023	Brazil	Meta-analysis
Gloy VL (57)	2013	Switzerland	Meta-analysis
Vasdeki D (58)	2022	Switzerland	Review Article
Frenken M (59)	2022	USA	Retrospective Cohort
Hage K (60)	2024	USA	Retrospective Cohort
Ghanem OM (61)	2024	USA	Retrospective Cohort
Aminian A (62)	2017	USA	Retrospective Cohort
Aron-Wisnewsky J (63)	2017	Germany	Retrospective Cohort
Craig Wood G (64)	2018	USA	Retrospective Cohort
Bechtel P (65)	2013	USA	Retrospective Cohort
Modasi A (66)	2019	USA	Retrospective Cohort
Abi Mosleh K (67)	2023	USA	Retrospective Cohort
Salame M (68)	2023	USA	Retrospective Cohort
Winder AA (69)	2018	USA	Meta-analysis
Aminian A (70)	2022	USA	Retrospective Cohort
Jawhar N (71)	2023	USA	Retrospective Cohort
Clapp B (72)	2022	USA	Meta-analysis
Maroun J (73)	2023	USA	Retrospective Cohort
Hefler J (74)	2019	USA	Retrospective Cohort
Sood A (76)	2016	Switzerland	Meta-analysis
Ghanem OM (77)	2024	USA	Review Article
Ghanem OM (78)	2024	USA	Review Article
Verhoeff K (79)	2021	USA	Retrospective Cohort
Fang Y (80)	2023	USA	Retrospective Cohort
Sureshkumar KK (81)	2021	USA	Retrospective Cohort
Lee Y (82)	2021	Canada	Meta-analysis
Haugen CE (83)	2019	USA	Retrospective Cohort
Chierici A (84)	2023	USA	Meta-analysis
Sharma I (85)	2023	USA	Retrospective Cohort
Theochari CA (86)	2018	USA	Meta-analysis
Ardila-Gatas J (87)	2019	USA	Retrospective Cohort
Ghanem OM (88)	2023	USA	Review Article
Fry BT (89)	2024	USA	Retrospective Cohort
Clarke-Pearson DL (90)	2013	USA	Review Article
Young MC (91)	2023	USA	Retrospective Cohort

Supplementary Table S1. Summary of the included studies
